# Abnormal lens thickening in a child with Weill–Marchesani syndrome 4: A 3-year follow-up case report

**DOI:** 10.3389/fmed.2022.1021489

**Published:** 2023-01-09

**Authors:** Junting Huang, Kailai Nie, Xinpin Lv, Yuting Liu, Guiqi Yang, Junjiang Fu, Longqian Liu, Hongbin Lv

**Affiliations:** ^1^Department of Ophthalmology, West China Hospital, Sichuan University, Chengdu, China; ^2^Research Laboratory of Ophthalmology and Vision Sciences, State Key Laboratory of Biotherapy, West China Hospital, Sichuan University, Chengdu, China; ^3^Department of Ophthalmology, The Affiliated Hospital of Southwest Medical University, Luzhou, China; ^4^Key Laboratory of Epigenetics and Oncology, The Research Center for Preclinical Medicine, Southwest Medical University, Luzhou, China

**Keywords:** Weill–Marchesani syndrome, *ADAMTS17* gene, lens, myopia, microspherophakia

## Abstract

**Background:**

Weill–Marchesani syndrome 4 (WMS4) is caused by *ADAMTS17* gene variant and clinical abnormalities including lenticular myopia, ectopia lentis, glaucoma, microspherophakia, brachydactyly, and short stature. Due to free of heart defects and joint stiffness compared with other WMS forms, WMS4 has an insidious onset and is often misdiagnosed as high myopia. We combined multiple imaging biometry and whole-exome sequencing to diagnose a case of WMS4 with a 3-year follow-up.

**Case presentation:**

An 8-year-old boy presented to our ophthalmology department with progressive myopia for 1 year. He had high myopia in both eyes with normal funds, intraocular pressure, and axial length. Ocular examination revealed thicker lenses (right 4.38 mm, left 4.31 mm) with a smaller equatorial diameter (right 7.33 mm and left 7.17 mm) compared to normal children of the same age. Finger length measurement indicates brachydactyly. Whole-exome sequencing identified compound heterozygous missense variants c.2984G > A (p.Arg995Gln) and c.2254A > G (p.Ile752Val) in the *ADAMTS17* gene. During the 3 years of follow-up, the thickness of lenses increased significantly (right 4.49 mm, left 4.48 mm), but the equatorial diameter of the lenses had no significant change (right 7.32 mm, left 7.21 mm). As the equivalent lens power increased, the patient’s myopia spherical refractive error rose accordingly. Although the anterior chamber angle remained open during follow-up, the intraocular pressure increased to right 20.4 mmHg and left 19.6 mmHg, Iridodonesis and short stature were present.

**Conclusion:**

This case report highlights the abnormal thickening of the lens in WMS4 compared to the physiological thinning process during childhood. Comprehensive clinical examinations and genetic testing may improve diagnosis, which allows early therapeutic interventions for complications and better visual outcomes for the patient.

## Introduction

Weill–Marchesani syndrome (WMS) is a rare genetic disease that mainly affects the development of the eye, musculoskeletal system, and cardiovascular system. The main phenotypes are short stature, joint stiffness, thickened skin, brachydactyly, and ocular anomalies including microspherophakia, high myopia, angle-closure glaucoma, and cataract (1, 2). The autosomal dominant form of WMS is caused by variants in the *FBN1* gene (WMS2) (3), whereas the autosomal recessive form is caused by variants in genes of *ADAMTS10* (WMS1) (4), *LTBP2* (WMS3) (5), and *ADAMTS17* (WMS4) (6). The main difference between WMS4 caused by the *ADAMTS17* gene variant and other WMS forms is that WMS4 is commonly free of heart defects and joint stiffness (7–9). Morales et al. (6) firstly reported *ADAMTS17* variants in humans with WMS4 and defined the character of the WMS4 clinical abnormalities including lenticular myopia, ectopia lentis, glaucoma, spherophakia, brachydactyly, and short stature. This disease is also rarely reported in China (7, 10).

In this report, a combination of multiple imaging tests and whole-exome sequencing has been performed to identify a WMS4 patient with microspherophakia, short stature, and brachydactyly in a Chinese family. Abnormal thickening of the lens of the patient was observed during a 3-year follow-up.

### Case report

**FIGURE 1:**
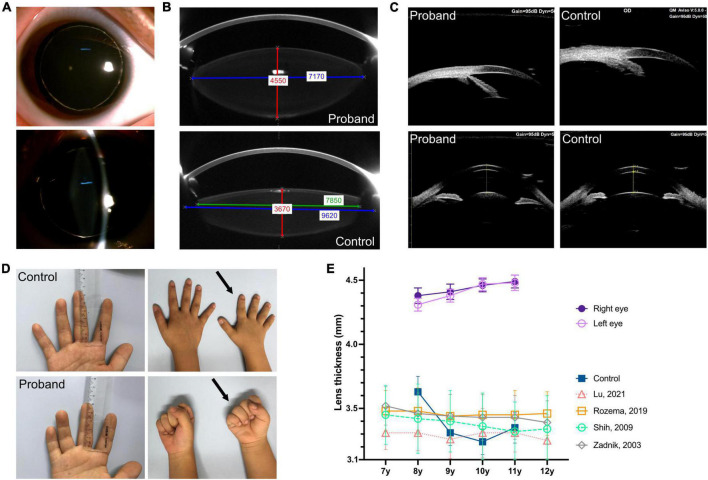
Clinical characteristics of eye, finger length, and hand joint. **(A)** Slit lamp examination after pupil dilatation completely reveals the equatorial region of the lens as well as bilateral microspherophakia. **(B)** PENTACAM shows that the lens of the proband is thicker than that of the emmetropic child in same age (control), red line: Thickness; the equatorial diameter was smaller, blue line: Actual equatorial diameter measurement of the patient’s lens and the presumed value in the control eye; green line: Measurable value in the control eye. **(C)** UBM shows the wide angle of the anterior chamber in the proband and emmetropic child (control) (upper, right eye); the curvature of the anterior lens capsule increased and bulged forward compared to the control (lower, right eye). **(D)** Middle finger length measurement indicates brachydactyly (5.1 cm, –2.4 standard deviation); the right hand of the proband (at age eight, arrows) show brachydactyly and the absence of hand joint stiffness (make a full fist) comparing to a control of the same age and sex. **(E)** The proband’s lens was thicker and thickened with age between 8 and 11 years of age, compared with emmetropic children (control) and measurements reported in the literature (16, 18, 21).

An 8-year-old boy first visited the ophthalmic clinic with the chief complaint of a progressive decrease in binocular vision for 1 year. He had been diagnosed as high myopia in other hospitals several times with fast myopia progression. A total of 1% cyclopentolate eye drops were administered before optometry, slit lamp, biometry, and fundus examinations. Slit lamp tests revealed bilateral microspherophakia and showed that the equatorial region of the lens was fully visible after pupil dilation (Figure 1A). The spherical refractive error was −8.25 diopter and −7.50 diopter in the right eye and left eye respectively, but fundus examinations including ultra-widefield retinal imaging and optical coherence tomography angiography did not identify any characteristic changes in high myopia such as posterior staphyloma, myopic maculopathy, choroidal neovascularization, and retinal detachment (11–13). IOLMaster 700 biometry showed that the axial length was 22.07 mm in the right eye and 22.01 mm left eye (Table 1), which were within mean ± 1.96 standard deviation (SD) of the normal reference range (14–20). While the lens thickness (right 4.38 mm, left 4.31 mm) was significantly greater than normal (Table 1) (16–18, 21, 22). PENTACAM illustrated visually that the lens of the proband was thicker than normal children (control) of the same age (Figure 1B, red line). Although it was difficult for PENTACAM to measure *in vivo* the equatorial diameter of the lens in normal subject after adequate pupil dilatation, the patient’s lens equatorial diameter (right 7.33 mm and left 7.17 mm) was seen to be smaller than that of normal control child (Figure 1B, blue line, presumed value 9.62 mm) and smaller than that of lenses at the same age measured *ex vivo* (23–26). The objective scatter index of OQAS II was 1.14 ± 0.20 in the right eye and 1.17 ± 0.09 in the left eye, suggesting no significant loss of lens transparency. Ultrasound biomicroscopy demonstrated a wide angle of the anterior chamber, the iris and ciliary body showed no obvious abnormalities, the curvature of the anterior lens capsule increased and bulged forward, and the gap between the lens equatorial region of the lens and ciliary body became larger (Figure 1C). Intraocular pressure was normal (right 16.7 mmHg, left 16.0 mmHg) and corneal topography analyses excluded keratoconus (Table 1). Moreover, the patient exhibited brachydactyly (5.1 cm, −2.4 SD) (27) but did not report hand joint stiffness (Figure 1D, arrows) and was not of short stature at 8 years old (height 124 cm, −1.6 SD). No structural anomalies were found in the cardiovascular system or abdomen in ultrasound scans. Given that the patient had not yet developed symptoms such as ocular hypertension or lens ectopia, spectacles were prescribed to correct the refractive error with regular follow-up. Best-corrected visual acuity was 20/25 in both eyes. Neither the patient’s parents nor sisters had myopia, and there were no abnormalities in intraocular pressure, slit lamp and mydriatic fundus examination.

**TABLE 1:** Ocular biometric measurements of the patient and comparison with reference values reported in the literature.

Parameters	Method	8 years old	9 years old	10 years old	11 years old
**Axial length (mm)**					
Patient’s (right/left)	IOLMaster	22.07/22.01	22.15/22.06	22.39/22.32	22.62/22.55
Lu et al. (14) (boys)	IOLMaster	23.28 ± 0.84	23.61 ± 0.86	23.88 ± 0.99	23.97 ± 1.00
Fledelius et al. (15) (males)	IOLMaster	23.22 ± 0.64	23.70 ± 0.78		23.44 ± 0.71
Rozema et al. (16)	Ultrasound	23.06 ± 0.68	23.15 ± 0.69	23.32 ± 0.72	23.43 ± 0.72
Shih et al. (17) (boys)	Ultrasound	23.27 ± 0.90	23.49 ± 0.89	23.79 ± 1.00	23.89 ± 1.00
Zadnik et al. (18) (boys)	Ultrasound	23.14 ± 0.81	23.40 ± 0.70	23.43 ± 0.80	23.54 ± 0.84
Larsen (19) (males)	Ultrasound	22.33 ± 0.51	22.43 ± 0.47	22.50 ± 0.47	22.70 ± 0.82
**Lens thickness (mm)**					
Patient’s (right/left)	IOLMaster	4.38/4.31	4.41/4.38	4.46/4.47	4.49/4.48
Lu et al. (21) (boys)	Lenstar	3.31 ± 0.14	3.26 ± 0.15	3.31 ± 0.19	3.31 ± 0.15
Rozema et al. (16)	Ultrasound	3.48 ± 0.18	3.44 ± 0.17	3.45 ± 0.17	3.45 ± 0.19
Shih et al. (17) (boys)	Ultrasound	3.42 ± 0.27	3.40 ± 0.24	3.36 ± 0.26	3.32 ± 0.23
Zadnik et al. (18) (boys)	Ultrasound	3.46 ± 0.16	3.44 ± 0.17	3.43 ± 0.18	3.43 ± 0.17
**Other eye component (right/left)**					
Central corneal thickness (μm)	IOLMaster	553/548	564/559	554/552	547/545
Anterior chamber depth (mm)	IOLMaster	2.81/2.86	2.69/2.77	2.72/2.80	2.69/2.76
White to white (mm)	IOLMaster	12.0/12.0	12.0/11.9	11.9/12.0	11.9/12.0
Mean keratometry (diopter)	IOLMaster	44.42/44.58	44.12/44.35	44.44/44.62	44.14/44.41

**FIGURE 2:**
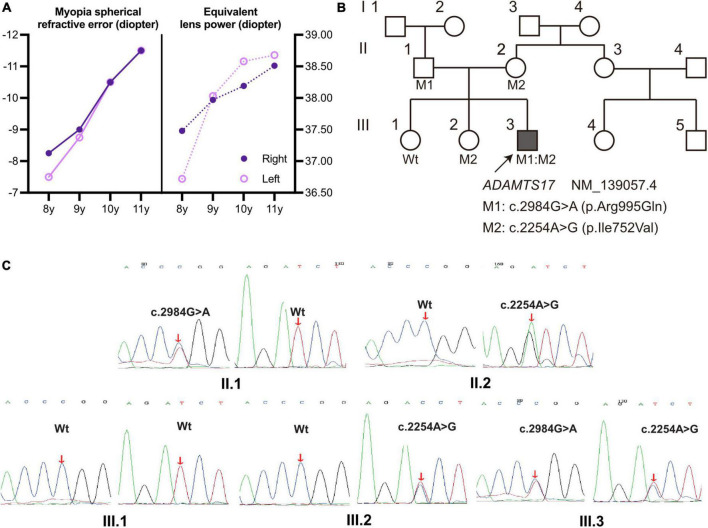
**(A)** The spherical refractive error of the proband increased (more negative) from 8 to 11 years of age and was more pronounced in the left eye, with the same trend as the calculated equivalent lens power (28); solid line: Myopia refractive error; dashed line: equivalent lens power. **(B)** Pedigree with WMS4; the proband III.3 is marked with a solid square and an arrow; unaffected individuals were labeled as blank symbols. Individuals with known genotypes were labeled, and carriers were labeled with the variants (M1:c.2984G > A, M2:c.2254A > G). **(C)** Sanger sequencing validated the compound heterozygous *ADAMTS17* variants in the WMS family. Red arrows indicate the variant sites.

During the subsequent 3 years of follow-up, the patient presented a progressive increase in lens thickness (Figure 1E and Table 1). The equivalent lens power of the patient was calculated (28) and showed a progressive increase, which was more pronounced in the left eye. It was consistent with the change in spherical refractive error (Figure 2A). In addition, the intraocular pressure of the patient was elevated to the upper limit of normal (right 20.4 mmHg, left 19.6 mmHg), and iridodonesis was observed (Supplementary Video). PENTACAM showed no significant increase in the equatorial diameter of the lens (right 7.32 mm and left 7.21 mm at 11 years of age) (23–26). At a recent follow-up height measurement, the patient (at age 10) exhibited short stature (height 131.2 cm, −2.7 standard deviation) (27).

Whole-exome sequencing was performed on the proband (Figure 2B; III.3, arrow) and compound heterozygous variants were successfully identified in exon 16 (c.2254A > G) and exon 21 (c.2984G > A) of *ADAMTS17* (NM_139057.4). The detailed result of whole exome sequencing was provided in the Supplementary Tables 1, 2. The variants of the *ADAMTS17* gene were confirmed by Sanger sequencing, and co-segregation analysis of the family was performed (Figure 2C). The result suggests that these compound heterozygous *ADAMTS17* variants and disease were co-segregated in this family and imply their potential role in the autosomal recessive WMS.

## Discussion

The human lens is almost spherical in early embryonic development and increasingly elliptical during the postnatal emmetropization process (29, 30). Measurements in school-age children between the ages of 6 and 10 years have shown that the axial dimension of the lens thins by an average of nearly 0.2 mm (22). Zadnik et al. (18) reported thickness thinning and flattening of the lens of 2,583 children aged 6–14 years. Data including 11,656 students indicated that the lens thinned within the 7–11 age range (17). A study included 596 Chinese children suggested a reduction in lens thickness and refractive lens power in children aged 6–13 years (21). Lens thinning at 7–10 years of age was also observed in a cohort of 303 emmetropic Singaporean children (16). However, the lens of the proband was thicker at 8 years of age than that of children of the same age and thickened by about 0.1 mm between 8 and 11 years old (Figure 1E and Table 1). Furthermore, lens thickness was generally thinner in myopes than emmetropes within 7–14 years of age, which may be a loss of equivalent lens power to compensate for the rapid axial growth of myopes (16, 17, 21, 31). In contrast, this WMS4 patient presented an abnormal lens thickening and a notable increase in equivalent lens power during the axial growth, resulting in progressive lens-induced high myopia.

In addition, the equatorial diameter of the patient’s lens did not increase significantly and was consistently lower than that of normal peers (24–26). Measurements of intact, clear, postmortem lenses have shown that the central thickness gradually increases after 20 years of age, while the equatorial diameter of the lens increases with life, with a particularly rapid increase in equatorial diameter before 20 years of age (24–26). As the equatorial diameter increases at nearly the same rate as the thickness, the relative shape of the lens is almost constant during adulthood (32). Thus, the lens thickness of children with WMS4 may not be thicker than that of adults (33), especially compared to elderly cataract patients (34). However, the lens thickness is still significantly thicker than that of normal children of the same age and the equatorial diameter is consistently smaller. Caution is needed when diagnosing microspherophakia in children of this age to avoid misdiagnosis.

The *ADAMTS17* gene locating on human chromosome 15q26.3, encodes a protease, which belongs to a superfamily of the ADAMTS, a disintegrin-like and metalloproteinase domain with thrombospondin-type 1 motifs and may participate in the regulation of assembly of microfibrils by modulating the fibrillin isoform (35). According to the gnomAD database, c.2984G > A (rs1289240183, dbSNP) is rare with a minor allele frequency of 0.00001. In contrast, c.2254A > G has not been reported by the 1,000 Genomes Project, the ExAC, gnomAD, or the NHLBI ESP database. These variants resulted in substitutions including isoleucine (Ile) to valine (Val) at amino acid 752 (p.Ile752Val, c.2254A > G), and arginine (Arg) to glutamine (Gln) at amino acid 995 (p.Arg995Gln, c.2984G > A) in the *ADAMTS17* protein (Supplementary Table 1). Two missense variants in *ADAMTS17* have been reported to be associated with WMS: p.Thr343Ala and p.Cys1023Tyr. Among them, the p.Thr343Ala variant resides in a zinc-dependent metalloprotease domain, which results in reduced secretion of *ADAMTS17* into the extracellular matrix (9). Evans et al. (8) first reported the p.Cys1023Tyr variant and revealed that a change in this amino residue results in significantly decreased secretion of *ADAMTS17*. Notably, the missense variants found in this case, p.Arg995Gln, and the nearby p.Cys1023Tyr are located in the same thrombospondin type 1 repeats domain. In addition, p.Ile752Val lies in the spacer domain, and splice variants of the spacer domain are reported to regulate proteolytic specificity and protein secretion of *ADAMTS17* (36).

Short stature and brachydactyly are obvious features of WMS4. Genome-wide association studies have shown that *ADAMTS17* is associated with human height (37), suggesting that it may play a role in human skeletal growth. Recently, Oichi et al. (38) established an *Adamts17* knockout mouse model, and found that mice recapitulate clinical phenotypes of WMS, including shorter long bones, brachydactyly, and thickened skin. They suggested that *ADAMTS17* modulates the bone morphogenetic protein (BMP) -Smad1/5/8 pathway possibly by inhibiting the incorporation of fibrillin-2 into microfibrils to regulate skeletal formation. This may explain the common phenotypes of short height and brachydactyly in WMS4 caused by *ADAMTS17* variants. Heart defects and joint stiffness are rarely seen in WMS4 that distinguish it from other WMS forms (Table 2) (7–9), these abnormalities were not found in this case.

**TABLE 2:** Ocular and systemic abnormalities in WMS with mutations in different genes.

WMS subtype (gene)	Ocular	Cardiovascular system	Musculoskeletal system	Pattern of inheritance
WMS1 (*ADAMTS10*) (4)	Microspherophakia, glaucoma, ectopia lentis, cataract	Cardiac anomalies	Short stature, joint stiffness, brachycephaly, brachydactyly, thick skin	Autosomal recessive
WMS2 (*FBN1*) (3)	Microspherophakia glaucoma ectopia lentis cataract	Cardiac anomalies	Short stature, joint stiffness, brachycephaly, brachydactyly, thick skin	Autosomal dominant
WMS3 (*LTBP2*) (5)	Ectopia lentis, increased intraocular pressure, microspherophakia	Cardiac anomalies	Short stature, joint stiffness, brachydactyly	Autosomal recessive
WMS4 (*ADAMTS17*) (6)	Ectopia lentis, glaucoma, microspherophakia	No	Short stature, brachydactyly	Autosomal recessive

The previous cases reported *ADAMTS17* variants lead to ectopia lentis and secondary glaucoma in WMS4 (6–10). *ADAMTS17* expression was detected by RNA *in situ* hybridization in multiple ocular tissues of embryonic mouse, including fiber cells of the lens equator, ciliary body epithelium at the zonule junction site, and trabecular meshwork (35). In addition, an *ADAMTS17* missense variant that causes primary lens luxation and primary open angle glaucoma in dogs has been reported (39). These results suggest a potential role of *ADAMTS17* in the lens zonule, trabecular meshwork and ciliary body. Therefore, the patient requires long-term clinical follow-up and timely management of possible complications to prevent optic nerve damage and visual field impairment due to secondary glaucoma. Early diagnosis is important in the management of WMS4, as possible complications including ectopia lentis, ocular hypertension, and glaucoma, may be intervened early.

In previous reports, patients with WMS4 usually had already developed peripheral iris synechiae, ectopia lentis, ocular hypertension, glaucoma, or had undergone surgical treatment at the time of initial diagnosis (6–10). This is because WMS4 has an insidious onset and is often diagnosed as high myopia with insidious early symptoms in the initial examination, especially in childhood. We made the diagnosis at an early stage and demonstrated changes in the biological parameters of the eyes during a long follow-up period, increasing the awareness of the disease. To avoid missed diagnosis and misdiagnosis, it is important to pay attention to the patients who have high myopia with narrow anterior chamber, without axial extension of the eye or myopic retinopathy. Awareness of the physiological developmental process of lens is needed. Comprehensive clinical examinations may improve diagnosis, and whole-exome sequencing has been frequently used to investigate genetic disorders which can be used to aid diagnosis.

## Author contributions

HL and LL: conception and design. LL: administrative support. YL and GY: provision of study materials or patients. JH and XL: collection and assembly of data. KN and JF: data analysis and interpretation and manuscript writing. All authors contributed to the article and approved the submitted version.
