# Supplementary material for: Abnormal lens thickening in a child with Weill–Marchesani syndrome 4: A 3-year follow-up case report
Source: Front Med (Lausanne). 2023 Jan 9;9:1021489. doi: 10.3389/fmed.2022.1021489 (PMC9868407; doi:10.3389/fmed.2022.1021489)
Supplement: Supplementary file 1 [file Table_1.DOCX]

| **Supplementary Table 1.** Characteristics of *ADAMTS17* variants and prediction of disease-causing effects | | | | | | | | |  |
| --- | --- | --- | --- | --- | --- | --- | --- | --- | --- |
| **Gene** | **Exon** | **Variants** | | | **In silico prediction** | | | | **ClinVar Accession** |
|  |  | **Nucleotide** | **Amino acid** | **Alleles (frequency^*^)** | | **SIFT** | **CADD** | **MSC** |  |
| *ADAMTS17*  NM_139057.4 | 21 | c.2984G>A | p.Arg995Gln | Het (0.00001) | | Damaging  (0.000) | Top 1% of deleterious variants (31) | High Impact (23.9) | VCV001210285.1 |
|  | 16 | c.2254A>G | p.Ile752Val | Het | | Damaging  (0.019) | Top 1% of deleterious variants (24.2) | High Impact (14.399) | VCV001210284.1 |
| ^*^Frequency: gnomAD allele frequency. Abbreviations: c, coding DNA reference sequence; p, protein reference sequence; Het, heterozygous; SIFT, sorting intolerant from tolerant; CADD, combined annotation dependent depletion; MSC, mutation significance cutoff. | | | | | | | | |  |

**Supplementary Table 2.** Whole exome sequencing findings of the proband.

| **Gene** | **Chromosomal location** | **Transcript** | **Mutation** | **Heterozygous/homozygous** | **Frequency** | **REVEL*** | **Pathogenicity analysis** | **Pattern of inheritance** |
| --- | --- | --- | --- | --- | --- | --- | --- | --- |
| C3 | chr19:6702476 | NM_0000  64;exon18 | c.2354+6G>C  (splicing) | het | 0.00160 | - | Uncertain | 1.AR  2.AD  3.- |
| C3 | chr19:6694650 | NM_0000  64;exon24 | c.2951-5T>C  (splicing) | het | 0.00280 | - | Uncertain | 1.AR  2.AD  3.- |
| PIEZO1 | chr16:88793562 | NM_0011  42864;exo n24 | c.3340C>G  (p.Q1114E) | het | 0.00800 | B | Uncertain | 1.AD 2.AR |
| VWA3B | chr2:98737874 | NM_1449  92;exon5 | c.655G>T  (p.E219X) | het | 0.00820 | - | Pathogenic | AR |
| VWA3B | chr2:98779397 | NM_1449  92;exon8 | c.1072G>A (p.V358M) | het | 0.01250 | B | Uncertain | AR |
| VPS11 | chr11:118939941118939941 | NM_0217  29;exon2 | c.220+2_220+  3insT  (splicing) | hom | 0.01920 | - | Uncertain | AR |
| CACNA1F | chrX:49082952 | NM_0051  83;exon11 | c.1415C>T (p.A472V) | hemi | 0.00900 | B | Uncertain | 1.XL  2.XLR  3.XL |
| RLIM | chrX:73812633 | NM_1833  53;exon5 | c.517C>G  (p.Q173E) | hemi | 0.00390 | B | Uncertain | XL |
| CACNA1S | chr1:201036040 | NM_0000  69;exon20 | c.2632G>A (p.V878M) | het | 0.00020 | D | Uncertain | 1.AD  2.AD  3.AD |
| GNAT1 | chr3:50231060 | NM_1444  99;exon4 | c.413G>A  (p.R138H) | het | - | D | Uncertain | 1.AR  2.AD  3.AD |
| MYO7A | chr11:76873966 | NM_0011  27180;exo n14 | c.1622C>G (p.P541R) | het | 0.00010 | D | Uncertain | 1.AR  2.AD  3.AR |
| NOTCH2 | chr1:120468219 | NM_0244  08;exon25 | c.4220C>T  (p.S1407L) | het | 0.00003 | B | Uncertain | 1.AD  2.AD |
| PBX1 | chr1:164529067 | NM_0025  85;exon1 | c.8A>G  (p.E3G) | het | - | B | Uncertain | AD |
| PKD1 | chr16:2161330 | NM_0010  09944;exo n15 | c.3838G>A (p.A1280T) | het | 0.00020 | B | Uncertain | AD |
| PLEC | chr8:1449987  61 | NM_2013  84;exon31 | c.5336G>A  (p.R1779H) | het | 0.00020 | B | Uncertain | 1.AR  2.AR  3.AR  4.AR  5.AD |
| POLG | chr15:89862259 | NM_0026  93;exon20 | c.3176A>G (p.N1059S) | het | 0.00020 | D | Uncertain | 1.AR  2.AR  3.AD  4.AR  5.AR |
| RORA | chr15:60797791 | NM_1342  61;exon6 | c.858A>C  (p.E286D) | het | - | D | Uncertain | AD |
| SMARCA2 | chr9:2186169 | NM_0030  70;exon32 | c.4535G>C (p.S1512T) | het | - | B | Uncertain | AD |
| TBX6 | chr16:30100446 | NM_0046  08;exon4 | c.439G>A  (p.D147N) | het | - | D | Uncertain | AD,A  R |
| TCF20 | chr22:42610107 | NM_0056  50;exon2 | c.1205G>A (p.S402N) | het | - | B | Uncertain | AD |
| THBS2 | chr6:169633085 | NM_0032  47;exon12 | c.1679C>T (p.P560L) | het | 0.00009 | D | Uncertain | - |
| MFAP5 | chr12:8804268-8804268 | NM_0034  80;exon7 | c.236dupA  (p.N79Kfs*9) | het | 0.00020 | - | Uncertain | AD |
| DCHS1 | chr11:6651241 | NM_0037  37;exon10 | c.4784A>G  (p.H1595R) | het | - | B | Uncertain | 1.AR  2.AD |
| PRDM16 | chr1:3331143 | NM_0221  14;exon10 | c.2623G>C (p.V875L) | het | - | B | Uncertain | AD |
| KCNK18 | chr10:118969242 | NM_1818  40;exon3 | c.587A>G  (p.D196G) | het | 0.00010 | B | Uncertain | - |
| IFITM5 | chr11:299453 | NM_0010  25295;exo n1 | c.38C>T  (p.P13L) | het | 0.00010 | B | Uncertain | AD |
| TSC2 | chr16:2138096 | NM_0005  48;exon40 | c.5116C>T  (p.R1706C) | het | 0.00080 | D | Uncertain | 1.- 2.-  3.AD |
| TOPORS | chr9:32542476 | NM_0058  02;exon3 | c.2047C>T (p.R683W) | het | 0.00040 | B | Uncertain | AD |
| AMPD3 | chr11:10514947 | NM_0010  25389;exo n7 | c.991C>T  (p.R331C) | het | 0.00310 | D | Uncertain | AR |
| LYST | chr1:235940508 | NM_0000  81;exon17 | c.5315A>G  (p.Q1772R) | het | 0.00060 | B | Uncertain | AR |
| DDX11 | chr12:31237908-31237911 | NM_0306  53;exon5 | c.487_489del  GAA  (p.163delE) | het | 0.00260 | - | Uncertain | AR |
| ETFB | chr19:51850299 | NM_0010  14763;exo n4 | c.725C>T (p.S242F) | het | 0.00870 | D | Uncertain | AR |
| HADHA | chr2:26420574 | NM_0001  82;exon14 | c.1465A>G (p.K489E) | het | 0.00170 | B | Uncertain | 1.AR  2.AR |
| PDE6B | chr4:658738 | NM_0002  83;exon18 | c.2193+5G>A  (splicing) | het | 0.00250 | - | Uncertain | 1.AR  2.AD |
| PRF1 | chr10:72360156 | NM_0010  83116;exo n2 | c.503G>A (p.S168N) | het | 0.00180 | B | Uncertain | 1.-  2.AR  3.- |
| PTPN14 | chr1:2145379 46 | NM_0054  01;exon18 | c.3344C>T  (p.P1115L) | het | 0.01850 | B | Uncertain | AR |
| GRHPR | chr9:37429747 | NM_0122  03;exon6 | c.512G>A  (p.R171H) | het | 0.01130 | D | Uncertain | AR |
| RAB3GAP2 | chr1:220445634 | NM_0124  14;exon1 | c.46G>A (p.A16T) | het | 0.00190 | B | Uncertain | 1.AR  2.AR |
| PCLO | chr7:82580387 | NM_0330  26;exon6 | c.9517C>T  (p.L3173F) | het | 0.00060 | B | Uncertain | AR |
| RNF216 | chr7:5781010 | NM_2071  11;exon4 | c.638G>C  (p.G213A) | het | 0.00630 | B | Uncertain | AR |
| CEP55 | chr10:95279565 | NM_0011  27182;exo n8 | c.1191+1G>C  (splicing) | het | - | - | Likely pathogen  ic | AR |
| FANCI | chr15:89805094 | NM_0011  13378;exo n6 | c.488C>A  (p.T163N) | het | 0.00060 | B | Uncertain | AR |
| FAM20C | chr7:299939 | NM_0202  23;exon10 | c.1748C>T (p.A583V) | het | 0.01110 | B | Uncertain | AR |
| COL18A1 | chr21:46888653 | NM_1304  44;exon2 | c.1849G>A (p.G617R) | het | 0.01420 | B | Uncertain | AR |
| KIAA1109 | chr4:123108615 | NM_0153  12;exon6 | c.575C>G (p.P192R) | het | 0.00600 | D | Uncertain | AR |
| CACNA2D4 | chr12:2022196 | NM_1723  64;exon3 | c.419C>T  (p.A140V) | het | 0.00430 | B | Uncertain | AR |
| RFX6 | chr6:117199067 | NM_1735  60;exon2 | c.332C>G  (p.T111R) | het | - | B | Uncertain | AR |
| SOS2 | chr14:50606781 | NM_0069  39;exon17 | c.2668-4T>C  (splicing) | het | 0.00120 | - | Uncertain | AD |
| HNF1B | chr17:36104563 | NM_0004  58;exon1 | c.313G>A  (p.E105K) | het | 0.00180 | D | Uncertain | 1.AD  2.-  3.AD |
| LAMA2 | chr6:129781361 | NM_0004  26;exon49 | c.6884G>A  (p.R2295H) | het | 0.00170 | B | Uncertain | 1.AR  2.AR |
| LAMA2 | chr6:129588259 | NM_0004  26;exon16 | c.2217G>T (p.W739C) | het | 0.01630 | B | Uncertain | 1.AR  2.AR |
| CEP290 | chr12:88444154 | NM_0251  14;exon53 | c.7186G>T  (p.D2396Y) | het | 0.01560 | B | Uncertain | 1.AR  2.AR  3.AR  4.AR  5.AR |
| CEP290 | chr12:88535085 | NM_0251  14;- | c.-1G>A  ((G-A) +909  to  transcription initiation site) | het | 0.00190 | - | Uncertain | 1.AR 2.AR  3.AR  4.AR  5.AR |
| SYNE1 | chr6:152621856 | NM_1829  61;exon93 | c.17602G>A (p.G5868R) | het | 0.00060 | B | Uncertain | 1.AD  2.AR  3.AR |
| SC5D | chr11:121175205 | NM_0069  18;exon3 | c.343+3A>G  (splicing) | het | 0.00680 | - | Uncertain | AR |
| SDHA | chr5:233687 | NM_0041  68;exon8 | c.991G>A  (p.A331T) | het | 0.00370 | D | Uncertain | 1.AR  2.-  3.AD  4.AR |
| PNPL A6 | chr19:7601165 | NM_006702;exon5 | c.198+6T>C (splicing) | het | - | - | Uncertain | 1.AR |
| ROBO3 | chr11:124744752 | NM_0223  70;exon13 | c.2020C>T (p.R674C) | het | 0.00150 | B | Uncertain |  |

*REVEL (rare exome variant ensemble learner) prediction: D: damage; B: benign; -: unknown.
